# A precise and consistent assay for major wall polymer features that distinctively determine biomass saccharification in transgenic rice by near-infrared spectroscopy

**DOI:** 10.1186/s13068-017-0983-x

**Published:** 2017-12-07

**Authors:** Jiangfeng Huang, Ying Li, Yanting Wang, Yuanyuan Chen, Mingyong Liu, Youmei Wang, Ran Zhang, Shiguang Zhou, Jingyang Li, Yuanyuan Tu, Bo Hao, Liangcai Peng, Tao Xia

**Affiliations:** 1 Biomass and Bioenergy Research Centre, Huazhong Agricultural University, Wuhan, 430070 China; 2 National Key Laboratory of Crop Genetic Improvement, Huazhong Agricultural University, Wuhan, 430070 China; 3 College of Plant Science and Technology, Huazhong Agricultural University, Wuhan, 430070 China; 4 College of Life Science and Technology, Huazhong Agricultural University, Wuhan, 430070 China; 5 Haikou Experimental Station, Chinese Academy of Tropical Agricultural Sciences, Haikou, 570102 China

**Keywords:** Transgenic plant, Rice, Biomass saccharification, Plant cell wall, Near infrared spectroscopy, Bioenergy

## Abstract

**Background:**

The genetic modification of plant cell walls has been considered to reduce lignocellulose recalcitrance in bioenergy crops. As a result, it is important to develop a precise and rapid assay for the major wall polymer features that affect biomass saccharification in a large population of transgenic plants. In this study, we collected a total of 246 transgenic rice plants that, respectively, over-expressed and RNAi silenced 12 genes of the *OsGH9* and *OsGH10* family that are closely associated with cellulose and hemicellulose modification. We examined the wall polymer features and biomass saccharification among 246 transgenic plants and one wild-type plant. The samples presented a normal distribution applicable for statistical analysis and NIRS modeling.

**Results:**

Among the 246 transgenic rice plants, we determined largely varied wall polymer features and the biomass enzymatic saccharification after alkali pretreatment in rice straws, particularly for the fermentable hexoses, ranging from 52.8 to 95.9%. Correlation analysis indicated that crystalline cellulose and lignin levels negatively affected the hexose and total sugar yields released from pretreatment and enzymatic hydrolysis in the transgenic rice plants, whereas the arabinose levels and arabinose substitution degree (reverse xylose/arabinose ratio) exhibited positive impacts on the hexose and total sugars yields. Notably, near-infrared spectroscopy (NIRS) was applied to obtain ten equations for predicting biomass enzymatic saccharification and seven equations for distinguishing major wall polymer features. Most of the equations exhibited high *R* ^2^/*R* ^2^_cv_/*R* ^2^_ev_ and RPD values for a perfect prediction capacity.

**Conclusions:**

Due to large generated populations of transgenic rice lines, this study has not only examined the key wall polymer features that distinctively affect biomass enzymatic saccharification in rice but has also established optimal NIRS models for a rapid and precise screening of major wall polymer features and lignocellulose saccharification in biomass samples. Importantly, this study has briefly explored the potential roles of a total of 12 *OsGH9* and *OsGH10* genes in cellulose and hemicellulose modification and cell wall remodeling in transgenic rice lines. Hence, it provides a strategy for genetic modification of plant cell walls by expressing the desired *OsGH9* and *OsGH10* genes that could greatly improve biomass enzymatic digestibility in rice.

**Electronic supplementary material:**

The online version of this article (10.1186/s13068-017-0983-x) contains supplementary material, which is available to authorized users.

## Background

Lignocellulose represents an enormous biomass resource for biofuels and chemical products. Food crops not only produce grains for human beings, but also provide large amounts of lignocellulose residues [1, 2]. In principle, biomass conversion involves three major steps: initial physical and chemical pretreatment for lignocellulose destruction, subsequent enzymatic hydrolysis to release fermentable sugars, and, finally, yeast fermentation to produce ethanol. However, lignocellulose recalcitrance leads to an unacceptably high cost for biofuel production [3]. To reduce recalcitrance, the genetic modification of plant cell walls has been proposed as a promising solution by selecting transgenic plants that over-express the key genes associated with cell wall biosynthesis and modification [4, 5]. It thus becomes essential to distinguish, among the transgenic plants, the major wall polymer features that basically determine biomass enzymatic saccharification.

Plant cell walls are mainly composed of cellulose, hemicellulose, and lignin with small amounts of pectin and wall proteins. Cellulose is a crystalline polymer composed of β-1,4-glucan chains, and its crystallinity has been characterized as the key feature that negatively affects enzymatic biomass saccharification in the plant species examined [5–7]. To reduce cellulose crystallinity, some researchers have considered selecting transgenic plants that over-express GH9 family genes, which encode glycoside hydrolase enzymes specific for β-1,4-glucan modification [2, 8, 9]. As xylan is the major hemicellulose in grass plants, the acetylation of xylan has been reported to negatively affect biomass saccharification and biofuel productivity by hindering the access cellulase enzymes to the cellulose surface and by producing acetic acid compounds that inhibit yeast fermentation [10, 11]. Furthermore, the degree of arabinose substitution of xylan has been shown to positively impact biomass enzymatic digestibility by reducing the cellulose crystalline index [12]. Since GH10 enzymes are involved in the modification of xylans and other hemicelluloses, over-expressing GH10 genes may alter the xylan structure to promote biomass saccharification in bioenergy crops [2].

Lignin is a phenylpropane wall polymer consisting of three major monomers: *p*-hydroxyphenyl (H), guaiacyl (G), and syringyl (S). The lignin is tightly associated with hemicelluloses to maintain plant mechanical strength and biomass recalcitrance, so it is thought to play a negative role in biomass saccharification [13]. More recently, lignin has been reported to play dual roles in biomass enzymatic hydrolysis, probably due to three distinctive monolignol proportions in genetic mutants and transgenic plants [14].

Rice (*Oryza sativa* L.) is one of the most important cereal crops around the world, and it also produces approximately 800 million metric tons of lignocellulose-based straws annually for potential bioethanol production [15]. To reduce lignocellulose recalcitrance, we have selected large-scale transgenic rice plants that, respectively, over-expressed seven *OsGH9* and five *OsGH10* family genes [2, 9, 16]. Since plant cell walls have complicated structures and dynamic networks, developing a precise and rapid approach to identify the key wall polymer features that could greatly enhance biomass saccharification among a large population of biomass samples remains a technical challenge. On the other hand, classic laboratory methods are time-consuming and costly for cell wall analysis.

Near-infrared spectroscopy (NIRS) has been applied as a non-destructive and rapid analytical tool to predict sample properties and component compositions. It is very efficient for high-throughput screening of a large population of samples at both qualitative and semi-quantitative levels. For instances, NIRS has been used for high-throughput phenotyping of multiple traits in crop breeding [17–19] and has also been applied to predict plant cell wall composition and biomass digestibility in different plant species [20–28]. However, due to limited variation in the normal cultivated species, little has been reported about the application of NIRS in a precise assay for both key wall polymer features and biomass enzymatic digestibility in rice.

In this work, we collected hundreds of transgenic rice samples that over-expressed and RNAi knocked-down a total of 12 typical *OsGH9* and *OsGH10* genes. Because those transgenic plants exhibited large variations in cell wall compositions and biomass saccharification, we established optimal equations for an excellent NIRS prediction of the major wall polymer features. Hence, our study provides a strategy for the genetic modification of plant cell walls by over-expressing or RNAi knocking-down *OsGH9* and *OsGH10* genes and demonstrated a precise and rapid NIRS assay, which may be applicable for large-scale screening of target traits in bioenergy crops and beyond.

## Results

### Large variations of cell wall composition in transgenic rice straws

**Fig. 1:**
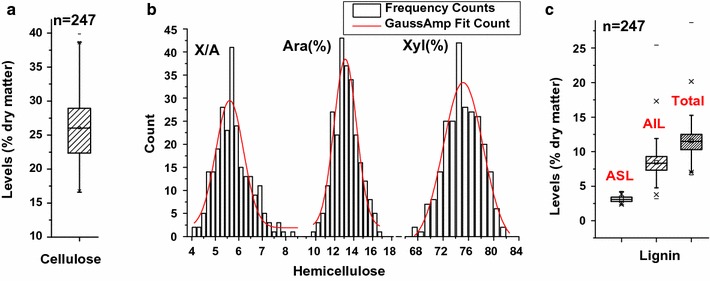
Variations of the wall polymer features in transgenic rice straws. **a** Cellulose levels (% dry matter). **b** Hemicellulosic monosaccharides (% total), arabinose (Ara), xylose (Xyl), xylose/arabinose ratio (X/A). **c** Lignin contents (% dry matter). *ASL* acid-soluble lignin, *AIL* acid-insoluble lignin

In this study, we collected and determined the cell wall compositions (cellulose, hemicelluloses, and lignin) of a total of 246 transgenic lines and one wild-type plant (Additional file 1: Table S1). As a result, a total of 247 rice straw samples exhibited large variations in three major wall polymer levels (Fig. 1). For comparison, the cellulose levels varied from 16.6 to 39.9% (Fig. 1a), whereas the levels of two major monosaccharides (Ara and Xyl) of hemicelluloses showed a perfectly normal data distributions (Fig. 1b). As Xyl/Ara (X/A) is the key parameter that inversely correlates with the degree of Ara substitution of xylan in rice, we also measured a normal distribution of X/A in a total of 247 samples. Furthermore, we detected that acid-insoluble lignin (AIL) showed much more variation than acid-soluble lignin (ASL), but the total lignin levels varied from 6.7 to 28.7% (Fig. 1c). Hence, these 246 transgenic rice plants presented a perfect sample population for the analysis of wall polymer features.

### Diverse biomass saccharification in transgenic rice straws

**Fig. 2:**
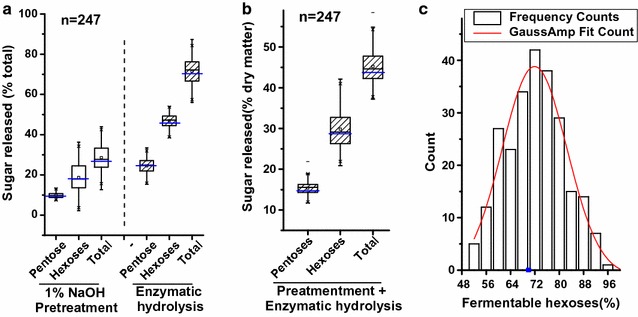
Variations of the biomass saccharification of transgenic rice straws. **a** Sugars released from the 1% NaOH pretreatment and the subsequent enzymatic hydrolysis (% total). **b** Total sugars released from the 1% NaOH pretreatment and enzymatic hydrolysis (% dry matter). **c** Fermentable hexoses only released from the enzymatic hydrolysis (% total hexoses). Blue lines in **a** and **b**, blue point in **c** wild-type

Biomass saccharification (digestibility) has been defined by measuring the yields of pentoses and hexoses released from physical and chemical pretreatment and subsequent enzymatic hydrolysis [25]. In this work, we detected diverse biomass saccharification in the 246 rice transgenic plants and one wild-type plant (Fig. 2). For comparison, the transgenic biomass samples showed large variations in the yields of pentoses and hexoses released either from 1% NaOH pretreatment or from enzymatic hydrolysis (Fig. 2a). Furthermore, the total yields of pentoses ranged from 11.7 to 21.9% after both pretreatment and enzymatic hydrolysis, whereas the yields of hexoses varied from 20.9 to 42.1%, leading to yields of total sugars (hexoses and pentoses) ranging from 37.2 to 58.4% among 247 samples (Fig. 2b). Because only the hexoses released from enzymatic hydrolysis are fermentable by yeast for ethanol production, we also found that the yields of fermentable hexoses showed a normal distribution ranging from 52.8 to 95.9% among the 247 biomass samples (Fig. 2c). Compared with the wild-type plant, several transgenic plants showed much higher biomass digestibility, especially for the fermentable hexoses, which even reached 95.9%. This also suggests that over-expressing or RNAi knocking-down *OsGH9* and *OsGH10* genes may be a potential genetic strategy for greatly improving biomass enzymatic saccharification in rice.

### Correlation between wall polymer features and biomass saccharification

**Fig. 3:**
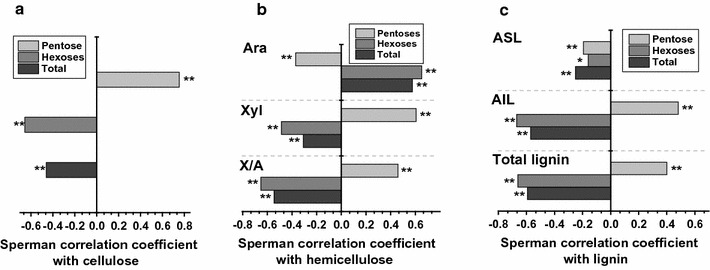
Correlations between the wall polymers and the biomass saccharification in transgenic rice straws (*n* = 247). **a** Correlation between cellulose levels and yields of sugars (pentoses, hexoses) released from both pretreatment and enzymatic hydrolysis. **b** Correlation between hemicellulose features and yields of sugars released. **c** Correlation between lignin features and yields of sugars released. *Ara* arabinose, *Xyl* xylose, *X/A* xylose/arabinose, *ASL* acid-soluble lignin, *AIL* acid-insoluble lignin. * and ** indicate significant correlations at the *p* < 0.05 and 0.01 levels

A correlation analysis has been applied that accounts well for the impact of wall polymer features on biomass enzymatic saccharification in different plant species [8, 12, 29]. In this study, we performed a Spearman correlation analysis among three major wall polymer features and the yields of sugars (pentoses, hexoses) released from pretreatment and enzymatic hydrolysis (Fig. 3). The cellulose levels showed a positive correlation with the yield of pentoses, but a negative correlation with the yields of hexoses and total sugars (Fig. 3a). For comparison, the Ara levels and the degree of Ara substitution (inverse X/A) exhibited significant positive correlations with the yields of hexoses and total sugars at *p* < 0.01 levels in the 246 transgenic rice plants and one wild-type plant (Fig. 3b), consistent with the previous reports in rice mutants [16]. However, both acid-soluble lignin (ASL) and acid-insoluble lignin (AIL) correlated negatively with the yield of hexoses (Fig. 3c), which differs from the previous findings in rice mutants. Significantly, all three wall polymer features showed significant correlations with the yields of pentoses, hexoses, or total sugars released by the pretreatment and subsequent enzymatic hydrolysis, suggesting that the polymer features could be employed to predict the biomass saccharification of transgenic rice plants.

### NIRS data in the transgenic rice population

**Fig. 4:**
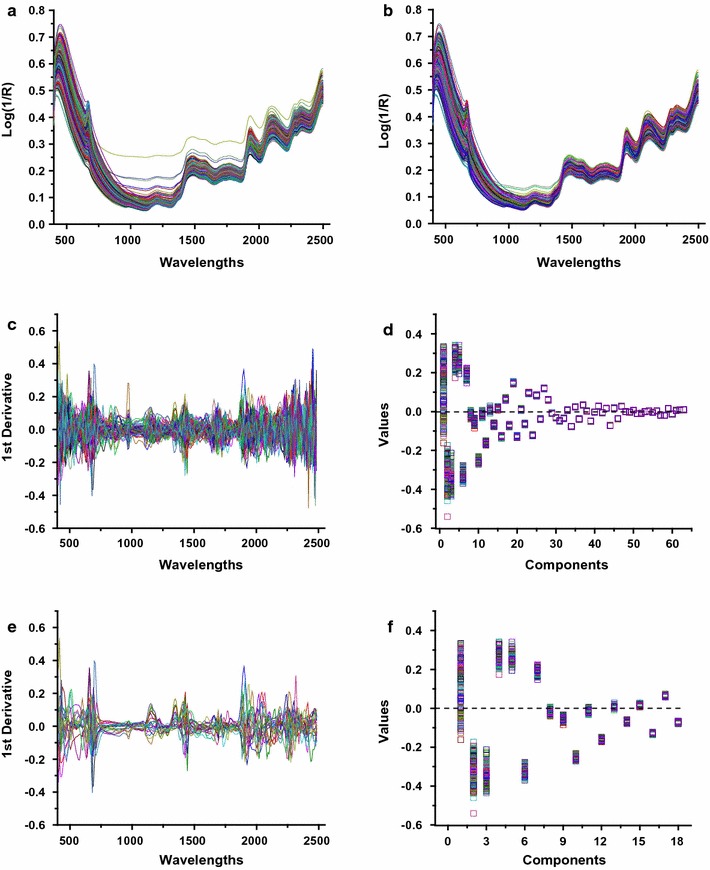
Characterization of the transgenic rice straws using near-infrared spectroscopy. **a** Original spectroscopy. **b** Selected spectroscopy after estimation of the GH outlier samples by PCA. **c**, **d** 63 principal components covering the globe variations. **e**, **f** Selected first 18 principal components explaining the variations to measure GH

NIRS data were collected in triplicate by recording the reflectance independently using an XDS Rapid Content™ Analyzer (FOSS, Co. LLC, Denmark). In general, the 247 averaged spectra were generated using the 246 transgenic rice plants and one wild-type plant (Fig. 4a). A principal component analysis (PCA) was carried out to eliminate the anomalous samples and reconstruct the spectral population (Fig. 4b). During the PCA process, the dimensionality of the spectral data was reduced by linearization processing of the original spectral data to generate new variables (principal components), which were orthogonal and uncorrelated to each other [30]. In this study, we generated a total of 63 new variables (principal components) that covered all of the variations in the original spectral population during the principal component analysis (Fig. 4c). The detailed variances of the spectral population on each principal component are presented in Fig. 4d. As a result, the first few components explained most of the variation, and little variation was observed due to component numbers over 30. Finally, only the first 18 of the 63 components were selected to measure the global H (GH), which explained 99.81% of the variation that characterizes the spectral population (Fig. 4e, f). Hence, seven samples were eliminated as GH outliers, leading to a narrow distribution of the spectroscopy of the remaining 240 samples (Fig. 4b).

### Calibration and validation sets for the NIRS modeling

**Fig. 5:**
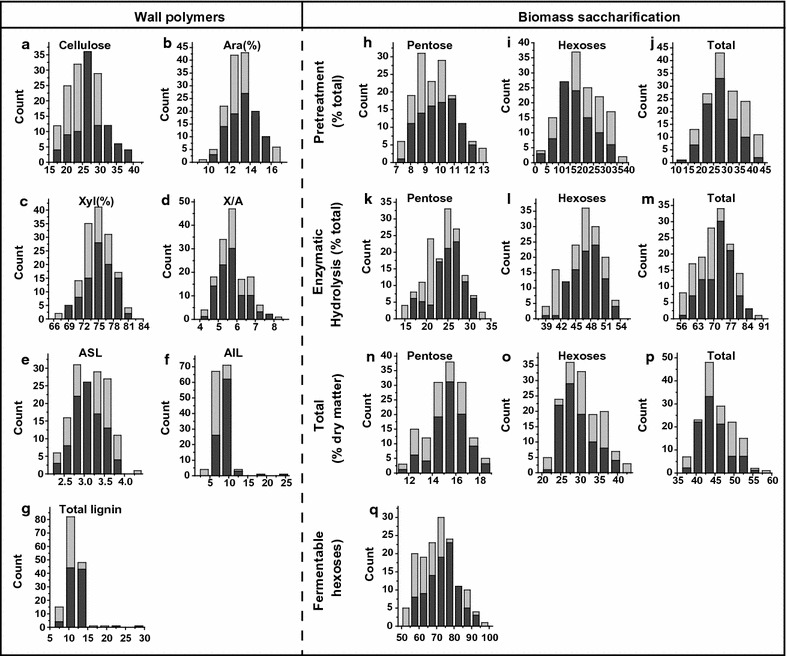
Distribution of calibration and validation sets for wall polymers and the relative biomass saccharification in transgenic rice straws. **a**–**g** Wall polymers (% dry matter), **a** cellulose; **b**–**d** hemicellulose, **b** arabinose (%), **c** xylose (%), **d** xylose/arabinose; **e**–**g** lignin, **e** acid-soluble lignin (ASL); **f** acid-insoluble lignin (AIL); **g** total lignin. **h**–**q** Biomass saccharification, **h**–**j** sugar released from the 1% NaOH pretreatment (% total), **h** pentose, **i** hexoses, **j** total; **k**–**m** sugar released from the enzymatic hydrolysis (% total), **k** pentose, **l** hexoses, **m** total; **n**–**p** total sugar released from the 1% NaOH pretreatment and the subsequent enzymatic hydrolysis (% dry matter), **n** pentose, **o** hexoses, **p** total; **q** fermentable hexoses (% total hexoses). Light gray: calibration sets (*n* = 147); dark gray: validation sets (*n* = 93)

A total of 240 samples were selected for modeling, based on the PCA analysis described above. Before calibration, 93 samples were randomly selected to form the external validation sets, and the remaining 147 samples were used for the calibration sets. A frequency distribution was obtained for the chemical values of the calibration and validation sets (Fig. 5). Most of them exhibited a normal distribution (except for AIL and total lignin), which was suitable for statistical analysis and the subsequent calibration. In addition, the calibration and validation sets were compared in terms of the mean, minimum, maximum, and standard deviation values (Additional file 2: Table S2). Therefore, the data demonstrated that the calibration and validation sets were comparable and reliable.

### NIRS modeling for wall polymer features

A principal component analysis (PCA) was further performed for the 147 samples in the calibration sets, and 15 components were obtained that accounted for 99.69% of the variation. The MPLS methods packed in the WinISI III software were applied for the calibration, and the standard error of calibration (SEC) and the coefficient determination of the calibration (*R* ^2^) were generated after this process. Furthermore, cross validations were performed to evaluate the calibration. During the cross validations, four groups were adopted by randomly selecting samples from the calibration sets into the cross-validation sets, leading to the standard error of the cross validations (SECV) and the coefficient determination of the cross validations (*R* ^2^_cv_). Moreover, the ratio performance deviation (RPD) was used to evaluate the predictive capacity of the equation [31]. Hence, the optimal equation could be selected on the basis of high *R* ^2^_cv_, *R* ^2^, and RPD values and low SEC, and SECV values.

Notably, all the equations for the prediction of wall polymer features exhibited high *R* ^2^_cv_, *R* ^2^ and RPD values, especially for the RPD values, which ranged from 1.73 to 2.79, indicating a high predictive capacity (Table 1). In detail, the equation for the acid-soluble lignin (ASL) showed the finest predictive capacity, based on its highest *R* ^2^_cv_, *R* ^2^, and RPD values among the calibrations of lignin content. The crystalline cellulose also showed a perfect calibration equation, with *R* ^2^_cv_ of 0.87 and an RPD of 2.79. Even though the major hemicellulosic monosaccharides (Xyl, Ara) showed relatively low *R* ^2^, *R* ^2^_cv,_ and RPD values, the X/A ratio had an RPD value of 1.98, which is sufficient for reasonable predictive capacity.

**Table 1:** Calibration statistics for equations generated for prediction of wall polymers in transgenetic rice straws

	Calibration									Cross validation		
	*N*	Terms	DT	SCM	Spectrum range (nm)	Mean	SD	SEC	*R* ^2^	SECV	*R* ^2^_cv_	RPD
Cellulose	140	3	2,4,4,2	SNVD	408–2492	25.11	4.45	1.55	0.88	1.59	0.87	2.79
Hemicellulose												
Ara (%)	145	5	1,4,4,1	SNV	408–2492	13.22	1.37	0.75	0.70	0.79	0.66	1.73
Xyl (%)	139	9	1,4,4,1	SNV	1108–2492	75.08	2.63	1.07	0.84	1.20	0.79	2.18
X/A	142	5	1,4,4,1	SNV	408–2492	5.75	0.77	0.37	0.77	0.39	0.75	1.98
Lignin												
ASL	141	9	1,4,4,1	MSC	1108–2492	3.13	0.41	0.12	0.91	0.15	0.87	2.78
AIL	140	5	1,4,4,1	SNV	408–2492	8.20	1.40	0.71	0.74	0.76	0.70	1.82
Total	140	9	0,0,1,1	WMSC	408–2492	11.33	1.55	0.69	0.80	0.75	0.77	2.07

*N* sample number, *Terms* number of principal component used for calibration, *DT* derivative treatment, *SCM* scatter correction methods, *SD* standard deviation of reference value, *SEC* standard error of calibration, *R* ^2^ determination coefficient, *SECV* standard error of cross validation, *R* ^2^_cv_, determination coefficient of cross validation, *RPD* ratio performance deviation (SD/SECV), *SNVD* a combination of standard normal variate and detrend, *SNV* standard normal variate, *MSC* standard multiple scatter, *WMSC* weighted multiple scatter correction, *Ara* arabinose, *Xyl* xylose, *X/A* xylose/arabinose, *ASL* acid soluble lignin, *AIL* acid insoluble lignin

**Fig. 6:**
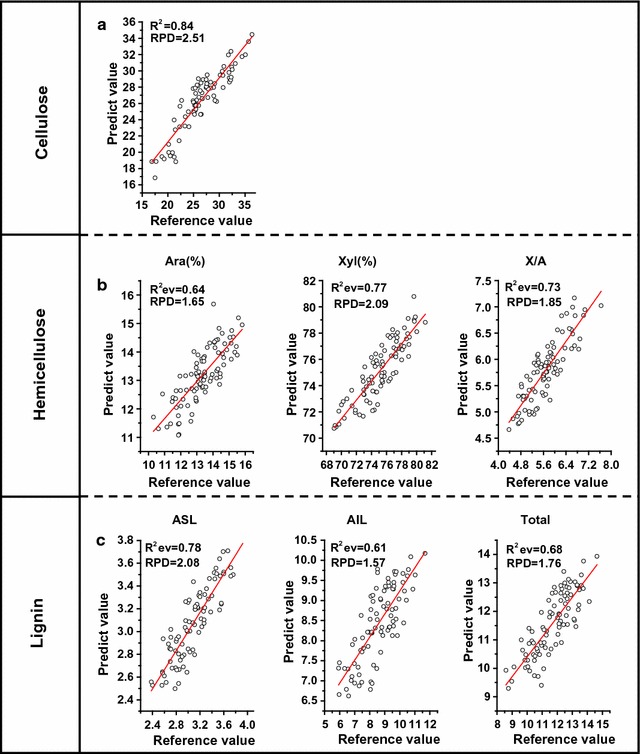
Correlations between the predicted and reference values for wall polymers. **a** Cellulose. **b** Hemicellulose. **c** Lignin. *Ara* arabinose, *Xyl* xylose, *X/A* xylose/arabinose, *ASL* acid-soluble lignin, *AIL* acid-insoluble lignin

Furthermore, the 93 randomly selected samples described above were used to evaluate the calibration equations by independent external validation. During the external validation, the coefficient determination of external validations (*R* ^2^_ev_) and the RPD remained the main factors for evaluating the calibration equation (Fig. 6). As a result, the equation for crystalline cellulose showed the highest *R* ^2^_ev_ value at 0.84 during the external validation, with an RPD value of the external validation at 2.51 (Fig. 6a), consistent with the calibration and cross-validation data. Notably, the equations for X/A and acid-soluble lignin (ASL), two major features for hemicelluloses and lignin, retained high *R* ^2^_ev_ and RPD values (Fig. 6b, c), suggesting that all the equations may be applicable for the prediction of wall polymer features.

### NIRS modeling for biomass saccharification

A similar calibration analysis was performed for the prediction of biomass saccharification in the transgenic rice plants. In general, the equations calibrated for biomass saccharification showed extremely high *R* ^2^ values ranged from 0.89 to 0.98 (Table 2), much higher than those of the wall polymer features described above. Meanwhile, the equations also showed relatively high *R* ^2^_cv_ values, ranging from 0.85 to 0.97 during cross validation, consistent with the calibration data. Notably, the RPD values of the cross validation reached from 2.56 to 5.89 (Table 2), indicating a perfect predictive capacity.

**Table 2:** Calibration statistics for equations generated for prediction of biomass saccharification in transgenetic rice straws

	Calibration									Cross validation		
	*N*	Terms	DT	SCM	Spectrum range (nm)	Mean	SD	SEC	*R* ^2^	SECV	*R* ^2^_cv_	RPD
Pretreatment (% total)												
Pentose	140	8	2,5,5,2	DET	1108–2492	9.64	1.26	0.37	0.91	0.42	0.89	2.97
Hexoses	142	10	1,4,4,1	SNV	780–2492	19.34	7.95	1.16	0.98	1.35	0.97	5.89
Total	141	10	2,4,4,2	SNVD	780–2492	29.26	6.81	1.00	0.98	1.38	0.96	4.95
Enzymatic hydrolysis (% total)												
Pentose	138	9	2,5,5,2	None	780–2492	23.90	4.01	0.69	0.97	0.83	0.96	4.80
Hexoses	143	10	2,10,10,2	None	780–2492	46.62	3.40	1.13	0.89	1.33	0.85	2.56
Total	141	10	2,4,4,2	SNVD	780–2492	70.74	6.81	1.00	0.98	1.38	0.96	4.95
Total sugar released (% dry matter)												
Pentose	141	8	2,10,10,2	DET	1108–2492	15.21	1.59	0.48	0.91	0.54	0.88	2.93
Hexoses	139	9	2,10,10,2	DET	780–2492	30.38	4.88	1.08	0.95	1.24	0.94	3.95
Total	138	9	1,4,4,1	WMSC^n^	1108–2492	45.31	3.97	1.21	0.91	1.38	0.88	2.88
Fermentable hexoses (% total hexoses)	144	9	2,8,8,2	WMSC	408–2492	71.17	10.11	1.78	0.97	2.20	0.95	4.60

*N* sample number, *Terms* number of principal component used for calibration, *DT* derivative treatment, *SCM* scatter correction methods, *SD* standard deviation of reference value, *SEC* standard error of calibration, *R* ^2^ determination coefficient, *SECV* standard error of cross validation, *R* ^2^_cv_ determination coefficient of cross validation, *RPD* ratio performance deviation (SD/SECV), *DET* detrend, *SNV* standard normal variate, *SNVD* a combination of standard normal variate and detrend, *WMSC* weighted multiple scatter correction

**Fig. 7:**
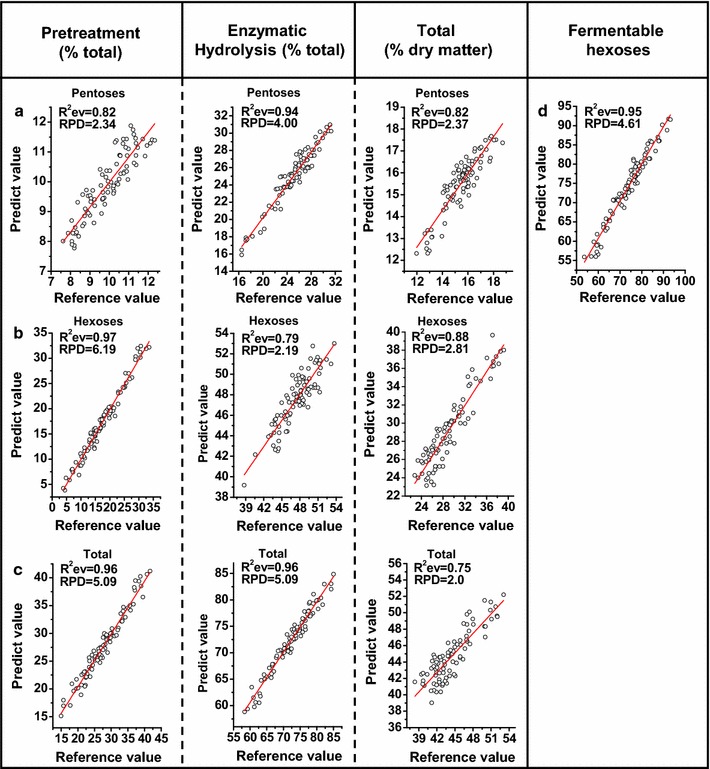
Correlations between the predicted and measured values for biomass saccharification. **a** Pentose yields, **b** hexose yields, **c** total sugar released, **d** fermentable hexoses (% total hexoses)

In addition, external validations were carried out to confirm the predictive capacity of the equation calibrated for biomass saccharification. All the equations showed a high correlation between the predicted values and the reference values measured by conventional analysis methods (Fig. 7). In particular, the yield of hexoses released from pretreatment showed an optimal *R* ^2^_ev_ value (Fig. 7b) compared to the yields of pentoses and total sugars released from both the pretreatment and the subsequent enzymatic hydrolysis (Fig. 7a, c). This was consistent with the calibration and cross-validation data with the highest *R* ^2^ and *R* ^2^_cv_ values at 0.98 and 0.97, respectively (Table 2). Notably, only the yield of fermentable hexoses obtained from enzymatic hydrolysis showed extremely high values for *R* ^2^ (0.97), *R* ^2^_cv_ (0.95), and *R* ^2^_ev_ (0.95) (Table 2 and Fig. 7d).

In conclusion, all the RPD values showed more than 2.0 values in both the cross validation and the independent external validation, with a highest RPD value of 6.19, indicating that NIRS is a precise and consistent assay for predicting biomass saccharification in the transgenic rice plants.

### Integrative calibrations for both wall polymers and biomass saccharification

An integrative calibration was performed by combining the calibration and validation sets as a new calibration set. As expected, the integrative calibrations were highly correlated with the previous calibrations, and some equations even showed much better parameters (Tables 1, 3). For instance, the equation for Ara (%) showed higher *R* ^2^, *R* ^2^_cv,_ and RPD values. However, some equations showed worse parameters, such as the equation for the yield of pentoses released from pretreatment, perhaps due to many samples showing close neighborhood H (NH) with much redundancy variation negatively affecting the calibration. Furthermore, the integrative calibration showed RPD values that ranged from 1.79 to 2.94 for wall polymer features, but retained high RPD values, from 2.39 to 6.63, for the prediction of biomass saccharification. Hence, the NIRS predictions should be applicable for both wall polymer features and biomass saccharification in transgenic rice plants and beyond.

**Table 3:** Calibration statistics for equations generated for prediction of wall polymers and biomass saccharification in transgenetic rice straws

	Calibration									Cross validation		
	*N*	Terms	DT	SCM	Spectrum range (nm)	Mean	SD	SEC	*R* ^2^	SECV	*R* ^2^_cv_	RPD
Wall polymers												
Cellulose	234	7	2,5,5,2	WMSC	1108–2492	26.09	4.95	1.56	0.9	1.69	0.88	2.94
Hemicellulose (% total)												
Ara (%)	236	5	2,4,4,1	None	780–2492	13.25	1.3	0.7	0.71	0.73	0.69	1.79
Xyl (%)	233	9	2,4,4,2	None	1108–2492	75.16	2.71	1.04	0.85	1.15	0.82	2.35
X/A	230	8	0,0,1,1	SNV	408–2492	5.73	0.72	0.36	0.75	0.37	0.73	1.94
Lignin (% dry matter)												
ASL	234	9	2,5,5,2	WMSC	780–2492	3.12	0.38	0.14	0.87	0.16	0.83	2.45
AIL	231	7	2,4,4,1	SNVD	1108–2492	8.34	1.34	0.69	0.74	0.74	0.69	1.81
Total	231	7	0,0,1,1	SNVD	408–2492	11.46	1.47	0.71	0.77	0.74	0.74	1.98
Biomass saccharification												
Pretreatment (% total)												
Pentose	234	7	2,4,4,2	MSC	1108–2492	9.78	1.24	0.43	0.88	0.47	0.86	2.63
Hexoses	228	8	2,8,8,1	SNV	1108–2492	18.6	7.58	1.08	0.98	1.14	0.98	6.63
Total	230	12	2,8,8,1	WMSC	780–2492	28.57	6.54	1.02	0.98	1.19	0.97	5.48
Enzymatic hydrolysis (% total)												
Pentose	230	11	2,10,10,2	None	1108–2492	24.37	3.84	0.74	0.96	0.81	0.96	4.74
Hexoses	233	10	1,4,4,1	DET	408–2492	46.94	3.19	1.15	0.87	1.26	0.85	2.54
Total	230	12	2,8,8,1	WMSC	780–2492	71.43	6.54	1.02	0.98	1.19	0.97	5.48
Total released sugar (% dry matter)												
Pentose	234	12	1,4,4,1	None	1108–2492	15.35	1.54	0.46	0.91	0.51	0.89	3.05
Hexoses	232	11	2,8,8,1	WMSC	1108–2492	29.89	4.62	1.2	0.93	1.4	0.91	3.3
Total	233	11	2,5,5,2	None	1108–2492	45.21	3.94	1.35	0.88	1.65	0.83	2.39
Fermentable hexoses (% total hexoses)	235	9	1,4,4,1	SNVD	408–2492	72.03	9.65	1.76	0.97	1.86	0.96	5.19

*N* sample number, *Terms* number of principal component used for calibration, *DT* derivative treatment, *SCM* scatter correction methods, *SD* standard deviation of reference value, *SEC* standard error of calibration, *R* ^2^ determination coefficient, *SECV* standard error of cross validation, *R* ^2^_cv_ determination coefficient of cross validation, *RPD* ratio performance deviation (SD/SECV), *WMSC* weighted multiple scatter correction, *SNV* standard normal variate, *SNVD* a combination of standard normal variate and detrend, *MSC* standard multiple scatter, *DET* detrend, *Ara* arabinose, *Xyl* xylose, *X/A* xylose/arabinose, *ASL* acid soluble lignin, *AIL* acid insoluble lignin

## Discussion

The genetic modification of plant cell walls has been considered to be a promising solution to addressing lignocellulose recalcitrance in bioenergy crops [4, 5]. In general, wall modification involves three strategies: altering the lignin polymer network, increasing the cellulose accessibility, and reducing inhibitors for biomass processing [5]. The genes of at least three groups of enzymes (RWA, AXY9, and TBR/TBL) and one BADH family have been identified for wall polysaccharide modification, in particular for xylan de-acetylation or de-feruloylation. Hence, these wall modifications could lead to either improving cellulase enzyme accessibility to enhance biomass saccharification or reducing the formation of inhibitors for increased ethanol fermentation by yeast [32–36]. In addition, other cell wall modification attempts have been made to enhance the yield of fermentable hexoses by carbon partitioning or glycosyl-transferase engineering [37–40]. However, because hundreds of genes have been reported to be involved in plant cell wall biosynthesis and modification, it remains difficult to identify the desired genes for transgenic plant selection towards enhancing biomass saccharification with little impact on plant growth in bioenergy crops [2, 41]. Recently, we have identified major wall polymer features that significantly affect biomass enzymatic digestibility using genetic mutants and natural germplasm accessions in rice, wheat, sweet sorghum, *Miscanthus,* and other grass plants [6–8, 12, 42]. Although both *OsGH9* and *OsGH10* family genes have been shown to play a major role in cellulose and hemicellulose modification, little is yet known about their enhancements to biomass saccharification in transgenic rice plants and other crops, probably due to the difficulty of selecting the desired genes for genetic manipulation.

More recently, we have selected a large population of transgenic rice plants by, respectively, over-expressing and RNAi silencing 12 representative genes from the *OsGH9* and *OsGH10* families (Additional file 1: Table S1). Using a total of 246 transgenic rice samples, this study has determined the significant variations in cell wall polymer features and in biomass saccharification, especially for fermentable hexoses, which even reached a highest yield of 95.9%. This suggests that over-expressing or RNAi knocking-down *OsGH9* and *OsGH10* genes may be a powerful genetic approach to greatly enhance biomass enzymatic saccharification in transgenic rice plants. Furthermore, using those greatly varied transgenic rice samples, we may be able to explore the biological functions and roles of the *OsGH9* and *OsGH10* families in cellulose and hemicellulose modification and cell wall remodeling in the future. More importantly, we were able to identify the desired *OsGH9* and *OsGH10* family genes for optimal genetic wall modification by screening out the transgenic rice lines that produce the highest biomass saccharification.

In addition, this work has examined a significant correlation between three wall polymer features and biomass saccharification (Fig. 3), indicating that the sample population of transgenic rice plants is sufficient to identify the key wall polymer features that determine biomass enzymatic hydrolysis. Surprisingly, even though the *OsGH9* and *OsGH10* families are not directly involved in lignin metabolism, this study showed that the lignin levels, including acid-soluble and acid-insoluble lignin, significantly varied, and found that the lignin levels were significantly correlated with biomass saccharification. This confirms that plant cell walls are dynamic networks and any small wall modification may lead to major wall polymer feature alteration. Therefore, it is important to develop a precise and rapid approach to screen for the desired genes that could greatly enhance biomass saccharification and slightly alter the wall polymer features in transgenic plants.

As a high-throughput screening method, NIRS has been applied to screen for “invisible” phenotypes (wall composition) in maize [19] and to predict cell wall compositions and biomass enzymatic digestibility in *Miscanthus* germplasm accessions and sweet sorghum mutants [20, 25, 27]. It has also been applied for the analysis of the chemical composition of rice straw [21], the quantification of the cell wall composition and monosaccharide content [22, 23], and the prediction of lignin syringyl/guaiacyl content [26]. However, the application of NIRS is critically limited by the calibration models, which in principle require large populations of varied samples to be reliable.

Rice (*O. sativa* L.) is one of the most important cereal crops around the world, but the variations in rice samples are restricted by the currently available rice cultivar species. Hence, this study initially attempted to collect rice samples that significantly varied by generating a total of 246 transgenic rice lines that, respectively, over-expressed and RNAi silenced a total of 12 *OsGH9* and *OsGH10* family genes. Due to the close association of the *OsGH9* and *OsGH10* families with cellulose and hemicellulose modifications (2, 9), we have observed large variations in the wall polymer features and biomass enzymatic saccharification among the transgenic rice samples.

Using this large population of varied transgenic rice samples, we have performed an NIRS assay for both biomass saccharification and three wall polymer features. Notably, most of the equations established based on the transgenic plants showed much higher *R* ^2^/*R* ^2^_cv_/*R* ^2^_ev_ and RPD values than those of the *Miscanthus* germplasm accessions and sweet sorghum genetic mutants. This indicates a precise NIRS assay in transgenic rice plants, probably due to more variation in the cell wall compositions and the biomass saccharification for transgenic biomass samples presented compared with the *Miscanthus* accessions and sweet sorghum mutants. Furthermore, this study not only established equations for ten parameters that account for biomass saccharification, but also generated equations for the prediction of seven wall polymer features, in particular the X/A ratio, a key wall polymer feature that determines the biomass saccharification in the grass plants examined. Hence, this study has established a precise and consistent NIRS assay for wall polymer features and biomass saccharification, which may be applicable for large-scale screening of transgenic rice plants.

## Conclusions

A total of 246 transgenic rice plants were selected by overexpressing and RNAi silencing 12 representative genes for cellulose and hemicellulose modification from the *OsGH9* and *OsGH10* families. Large variations in the cell wall compositions and the biomass saccharification were evaluated in the transgenic rice plants. In particular, the fermentable hexoses ranged from 52.8 to 95.9%, suggesting a potential wall modification strategy to greatly enhance biomass saccharification. Notably, the transgenic rice plants presented a perfect normal distribution of biomass samples, which was applicable for the NIRS analysis. Ten equations were ultimately generated for the prediction of biomass saccharification, and seven equations were applied for the key wall polymer feature analysis. Due to extremely high *R* ^2^/*R* ^2^_cv_/*R* ^2^_ev_ and RPD values in most of the equations, this study has demonstrated a precise and consistent NIRS assay for the large-scale screening of transgenic bioenergy crops and other bioenergy crops.

## Methods

### Plant materials

A total of seven *OsGH9* and five *OsGH10* genes were transformed into *Nipponbare* (NPB) using overexpressing and RNAi silencing constructs (Additional file 1: Table S1). A total of 246 transgenic lines and one wild-type plant (NPB) were harvested from the experimental field of Huazhong Agriculture University (Wuhan), and the mature stem tissues were collected and dried at 50 °C. The dried tissues were ground through 40 mesh and stored in a dry container until use.

### Plant cell wall polymer determination

The dried biomass samples were extracted twice with acetic-nitric acid–water (8:1:2) at 100 °C for 1 h. After centrifugation at 3000×*g* for 10 min, the remaining residues were washed five times with distilled water and collected as the cellulose fraction. The cellulose samples were dissolved in 72% sulfuric acid (w/w) and the total hexoses were determined using the anthrone/H_2_SO_4_ method described below. Three biological triplicates were performed for each sample.

Lignin was assayed using a two-step acid hydrolysis method. The samples were hydrolyzed with 72% (w/w) sulfuric acid at 30 °C for 90 min with gentle shaking at 115 rpm, and subsequently diluted to 3.97% (w/w) with distilled water and heated at 115 °C for 60 min. The supernatant liquids were measured at 205 nm for acid-soluble lignin (ASL), and the remaining residues were placed in a muffle furnace at 575 ± 25 °C for 4 h for the acid-insoluble lignin (AIL) assay [43]. For the determination of hemicellulosic monosaccharides, the acid-soluble supernatant for the acid-soluble lignin determination described above was used for the monosaccharide determination by GC–MS as described by Li et al. [12].

### Determination of biomass saccharification

The chemical pretreatments and the subsequent enzymatic hydrolysis were performed as previously described by Huang et al. [25] with minor modifications. The biomass sample was added to 6 mL of 1% NaOH (w/v). The sample tube was shaken at 150 rpm for 2 h at 50 °C, and centrifuged at 3000×*g* for 5 min. The supernatant was collected for determination of the total sugars released from the alkali pretreatment, and the pellet was washed five times with 10 mL distilled water and washed once with 10 mL of mixed cellulase reaction buffer (0.2 M acetic acid–sodium acetate, pH 4.8). Then, the samples were added to 6 mL of 0.16% (w/v) mixed-cellulases (containing ≥ 6 × 10^4^ U of β-glucanase, ≥ 600 U of cellulase, and ≥ 10 × 10^4^ U of xylanase from Imperial Jade Biotechnology Co., Ltd. Ningxia 750002, China). During the enzymatic hydrolysis, the samples were shaken at 150 rpm for 48 h at 50 °C. After centrifugation at 3000×*g* for 10 min, the supernatant was collected for the total sugar determination.

### Colorimetric assay of total hexoses and pentoses

A UV/VIS spectrometer (Shanghai MAPADA Instruments Co., Ltd. V-1100D) was used for the absorbance reading. Hexoses were detected by the anthrone/H_2_SO_4_ method [44] and pentoses were detected by the orcinol/HCl method [45]. Anthrone was purchased from Sigma-Aldrich Co. LLC. Ferric chloride and orcinol were purchased from Sinopharm Chemical Reagent Co., Ltd. The standard curves for hexoses and pentoses were obtained using d-glucose and d-xylose (purchased from Sinopharm Chemical Reagent Co., Ltd.), respectively.

### NIRS calibration and data acquisition

The near-infrared spectral data collection was performed using an XDS Rapid Content™ Analyzer (FOSS, Co. LLC, Denmark) as described by Huang et al. [25]. The WinISI III software package (Version 1.50e, Infrasoft International LLC) was used for the calibration and the acquisition of data as described by Huang et al. [25] with minor modifications.

Briefly, a principal component analysis (PCA) was carried out to structure and assess the variability of the spectral population before calibration. The GH outlier (GH > 3.0) samples were eliminated after the PCA. The modified partial least squares (MPLS) method was performed to provide a prediction equation. In MPLS, the near-infrared spectra residuals at each wavelength, obtained after calculating each factor, were standardized (divided by the standard deviations of the residuals at a wavelength) before calculating the next factor [46]. During calibration, eight derivative treatments were used: “0,0,1,1”, “1,4,4,1”, “2,4,4,1”, “2,4,4,2”, “2,5,5,2”, “2,8,8,1”, “2,8,8,2”, and “2,10,10,2”, where the first digit is the number of the derivative, the second is the gap over which the derivative is calculated, the third is the number of the first smoothing, and the fourth is the number of the second smoothing. Five scatter correction methods were provided, namely, standard normal variate (SNV), detrend only (DET), standard multiple scatter correction (MSC), a combination of SNV and DET (SNVD), and weighted multiple scatter correction (WMSC) to remove artifacts and imperfections from the data. Three wavelength ranges (408–2492, 780–2492, and 1108–2492 nm) were selected to obtain the best calibration equation.

## Additional files

**Additional file 1: Table S1.** Counts for the transgenetic lines in rice.

**Additional file 2: Table S2.** Calibration and validation sets for wall polymers (% dry matter) and biomass saccharification in transgenetic rice straws.

## Abbreviations

NIRS: near-infrared spectroscopy
Ara: arabinose
Xyl: xylose
X/A: xylose/arabinose
AIL: acid-insoluble lignin
ASL: acid-soluble lignin
PCA: principal component analysis
SEC: standard error of calibration
*R*^2^: coefficient determination of the calibration
SECV: standard error of cross-validation
*R*^2^_cv_: coefficient determination of cross-validation
RPD: ratio performance deviation
*R*^2^_ev_: coefficient determination of external-validation
SNV: standard normal variate
DET: detrend
MSC: standard multiple scatter
SNVD: a combination of standard normal variate and detrend
WMSC: weighted multiple scatter correction
