# Supplementary material for: A precise and consistent assay for major wall polymer features that distinctively determine biomass saccharification in transgenic rice by near-infrared spectroscopy
Source: Biotechnol Biofuels. 2017 Dec 7;10:294. doi: 10.1186/s13068-017-0983-x (PMC5719720; doi:10.1186/s13068-017-0983-x)
Supplement: Supplementary file 1 — Additional file 1: Table S1. Counts for the transgenetic lines in rice. [file 13068_2017_983_MOESM1_ESM.ppt]

## Slide 1
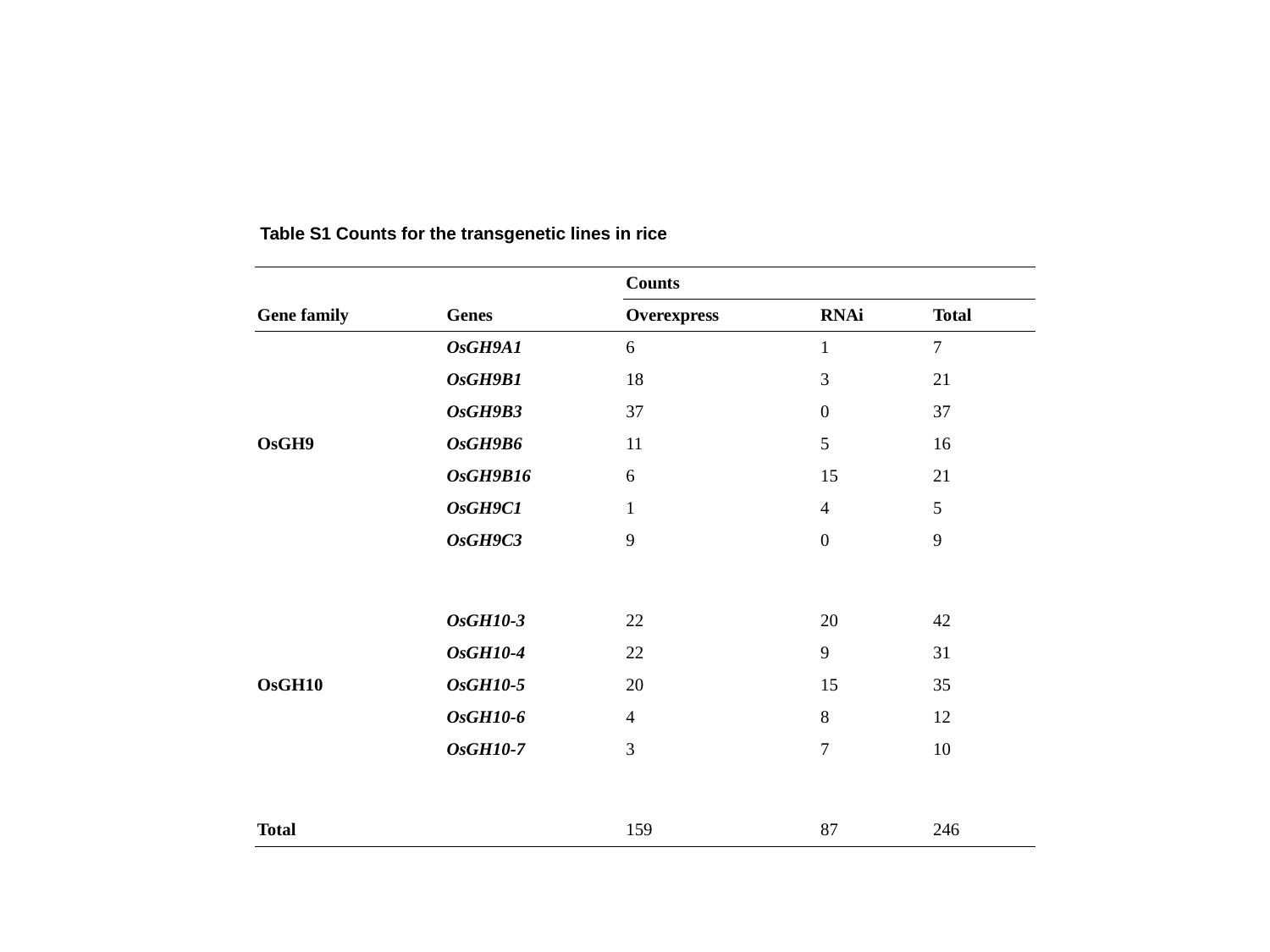

Table S1 Counts for the transgenetic lines in rice
| | | Counts | | |
| --- | --- | --- | --- | --- |
| Gene family | Genes | Overexpress | RNAi | Total |
| OsGH9 | OsGH9A1 | 6 | 1 | 7 |
| | OsGH9B1 | 18 | 3 | 21 |
| | OsGH9B3 | 37 | 0 | 37 |
| | OsGH9B6 | 11 | 5 | 16 |
| | OsGH9B16 | 6 | 15 | 21 |
| | OsGH9C1 | 1 | 4 | 5 |
| | OsGH9C3 | 9 | 0 | 9 |
| | | | | |
| OsGH10 | OsGH10-3 | 22 | 20 | 42 |
| | OsGH10-4 | 22 | 9 | 31 |
| | OsGH10-5 | 20 | 15 | 35 |
| | OsGH10-6 | 4 | 8 | 12 |
| | OsGH10-7 | 3 | 7 | 10 |
| | | | | |
| Total | | 159 | 87 | 246 |
