# Supplementary material for: A precise and consistent assay for major wall polymer features that distinctively determine biomass saccharification in transgenic rice by near-infrared spectroscopy
Source: Biotechnol Biofuels. 2017 Dec 7;10:294. doi: 10.1186/s13068-017-0983-x (PMC5719720; doi:10.1186/s13068-017-0983-x)
Supplement: Supplementary file 2 — Additional file 2: Table S2. Calibration and validation sets for wall polymers (% dry matter) and biomass saccharification in transgenetic rice straws. [file 13068_2017_983_MOESM2_ESM.ppt]

## Slide 1
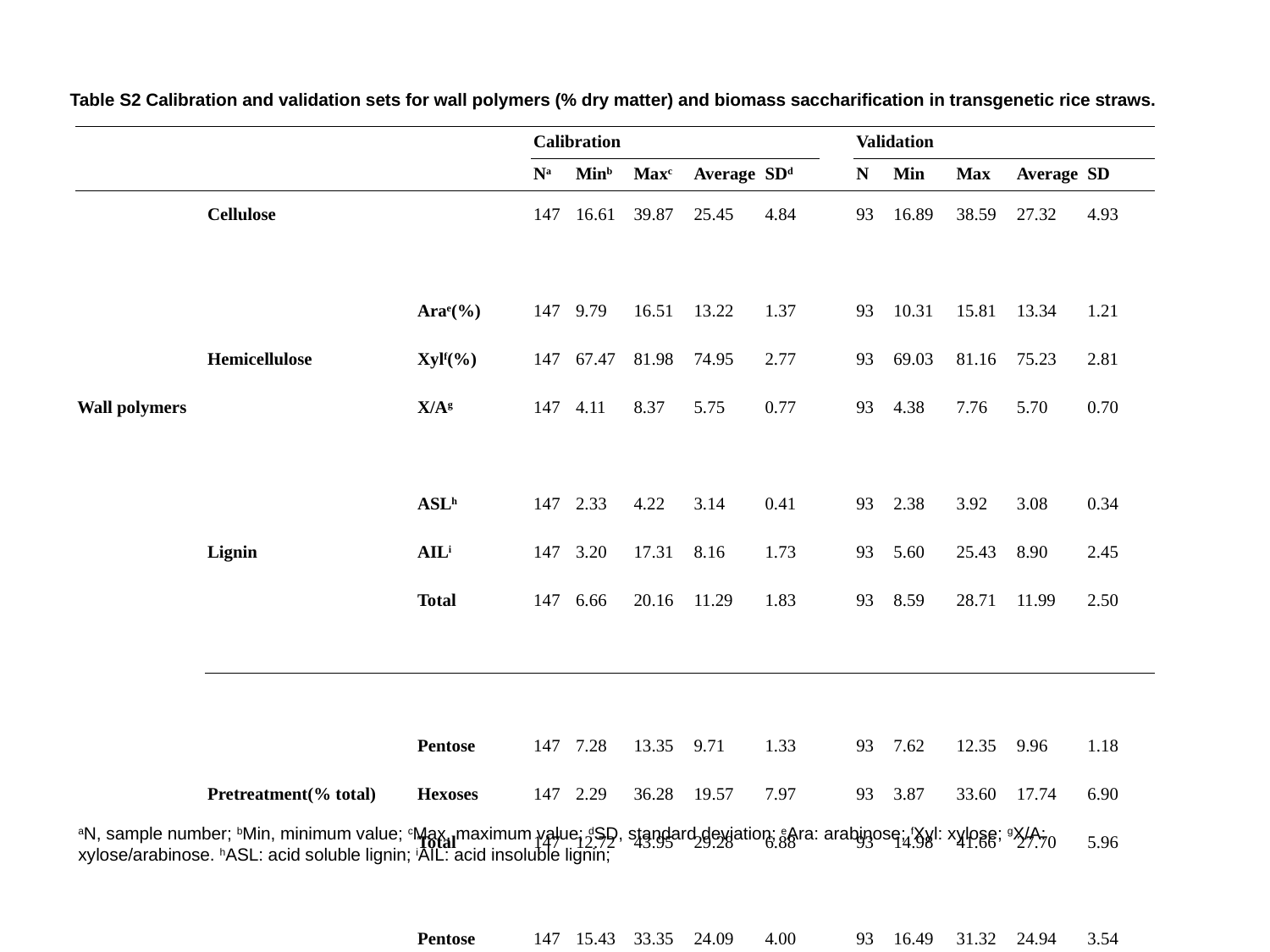

Table S2 Calibration and validation sets for wall polymers (% dry matter) and biomass saccharification in transgenetic rice straws.
| | | | Calibration | | | | | | Validation | | | | |
| --- | --- | --- | --- | --- | --- | --- | --- | --- | --- | --- | --- | --- | --- |
| | | | Na | Minb | Maxc | Average | SDd | | N | Min | Max | Average | SD |
| Wall polymers | Cellulose | | 147 | 16.61 | 39.87 | 25.45 | 4.84 | | 93 | 16.89 | 38.59 | 27.32 | 4.93 |
| | | | | | | | | | | | | | |
| | Hemicellulose | Arae(%) | 147 | 9.79 | 16.51 | 13.22 | 1.37 | | 93 | 10.31 | 15.81 | 13.34 | 1.21 |
| | | Xylf(%) | 147 | 67.47 | 81.98 | 74.95 | 2.77 | | 93 | 69.03 | 81.16 | 75.23 | 2.81 |
| | | X/Ag | 147 | 4.11 | 8.37 | 5.75 | 0.77 | | 93 | 4.38 | 7.76 | 5.70 | 0.70 |
| | | | | | | | | | | | | | |
| | Lignin | ASLh | 147 | 2.33 | 4.22 | 3.14 | 0.41 | | 93 | 2.38 | 3.92 | 3.08 | 0.34 |
| | | AILi | 147 | 3.20 | 17.31 | 8.16 | 1.73 | | 93 | 5.60 | 25.43 | 8.90 | 2.45 |
| | | Total | 147 | 6.66 | 20.16 | 11.29 | 1.83 | | 93 | 8.59 | 28.71 | 11.99 | 2.50 |
| | | | | | | | | | | | | | |
| | | | | | | | | | | | | | |
| Biomass saccharification | Pretreatment(% total) | Pentose | 147 | 7.28 | 13.35 | 9.71 | 1.33 | | 93 | 7.62 | 12.35 | 9.96 | 1.18 |
| | | Hexoses | 147 | 2.29 | 36.28 | 19.57 | 7.97 | | 93 | 3.87 | 33.60 | 17.74 | 6.90 |
| | | Total | 147 | 12.72 | 43.95 | 29.28 | 6.88 | | 93 | 14.98 | 41.66 | 27.70 | 5.96 |
| | | | | | | | | | | | | | |
| | Enzymatic hydrolysis(% total) | Pentose | 147 | 15.43 | 33.35 | 24.09 | 4.00 | | 93 | 16.49 | 31.32 | 24.94 | 3.54 |
| | | Hexoses | 147 | 38.59 | 53.93 | 46.62 | 3.41 | | 93 | 38.74 | 53.72 | 47.37 | 2.87 |
| | | Total | 147 | 56.05 | 87.28 | 70.72 | 6.88 | | 93 | 58.34 | 85.02 | 72.30 | 5.96 |
| | | | | | | | | | | | | | |
| | Total released sugar (% dry matter) | Hexoses | 147 | 20.89 | 42.12 | 30.27 | 4.81 | | 93 | 22.79 | 39.20 | 29.24 | 4.22 |
| | | Pentose | 147 | 11.73 | 18.89 | 15.22 | 1.59 | | 93 | 11.97 | 18.72 | 15.48 | 1.38 |
| | | Total | 147 | 37.19 | 58.44 | 45.49 | 4.17 | | 93 | 38.34 | 54.04 | 44.71 | 3.62 |
| | | | | | | | | | | | | | |
| | Fermentable hexoses (% total hexoses) | | 147 | 52.82 | 95.93 | 71.16 | 10.01 | | 93 | 53.55 | 93.27 | 73.31 | 8.75 |
aN, sample number; bMin, minimum value; cMax, maximum value; dSD, standard deviation; eAra: arabinose; fXyl: xylose; gX/A: xylose/arabinose. hASL: acid soluble lignin; iAIL: acid insoluble lignin;
